# Innovative use of therapeutic antibodies in lung cancer: the current landscape of ADCs and other antibody-based therapies

**DOI:** 10.3389/fonc.2026.1814095

**Published:** 2026-07-08

**Authors:** Matthew Lee, Christine M. Lovly

**Affiliations:** Department of Medical Oncology, City of Hope Comprehensive Cancer Center, Duarte, CA, United States

**Keywords:** antibody-drug conjugate (ADC), non-small cell lung cancer, precision oncology, targeted drug delivery, tumor microenvironment, bispecific antibodies

## Abstract

The therapeutic landscape for non-small cell lung cancer (NSCLC) and small cell lung cancer (SCLC) has been profoundly transformed by the advent of novel antibody therapies. These include antibody-drug conjugates (ADCs) and various novel iterations. Each therapy is at a different stage of clinical development, with several agents now approved for clinical use, others in late-stage trials, and a robust pipeline of preclinical candidates. This review will provide an overview of the antibody-based therapies, focusing on their molecular targets and clinical context in lung cancer and the future of this rapidly emerging field.

## 1 Introduction

Lung cancer remains the leading cause of cancer-related mortality, underscoring a persistent need for therapeutic strategies that extend beyond conventional chemotherapy, molecularly targeted small molecules, and immune checkpoint inhibitors. While monoclonal antibodies have transformed the treatment landscape, their efficacy is often limited and curtailed by variable antigen and target expression, suboptimal intra-tumoral penetration, immune evasion/resistance, and toxicities. These limitations have catalyzed the development of antibody platforms that deliver cytotoxic payloads, activate immune effector mechanisms, or provide conditional tumor-selective activation.

Recent advances in antibody engineering have catalyzed the emergence of next-generation therapeutic platforms that move beyond simple target blockades to actively deliver cytotoxic agents or redirect immune effector cells. Among these, antibody–drug conjugates (ADCs) have rapidly gained prominence in lung cancer by combining precise antigen recognition with the potency of highly cytotoxic payloads. In parallel, complementary antibody-based modalities—including bispecific antibodies and bispecific ADCs, probody-drug conjugates (PDCs), immune-stimulating ADCs (ISAC), protein-degrader ADCs (DACs), biparatopic ADCs, bispecific and trispecific T-cell engagers (BiTEs and TriTEs), radioligand/radioimmunotherapeutic antibodies, and nanobody-based constructs—are expanding the functional versatility of therapeutic antibodies.

Together, these innovative platforms exemplify a potential paradigm shift in lung cancer treatment, wherein antibodies serve as modular delivery vehicles and as immune interfaces, allowing more direct impact on tumor cells rather than passive binding agents, with the potential to improve clinical outcomes. This review highlights the design principles, clinical progress, and translational challenges of these emerging antibody-based therapies, with a particular emphasis on ADCs, and situates them within the evolving landscape of precision oncology for lung cancer.

## 2 ADCs: more than targeted chemotherapy?

**Figure 1:**
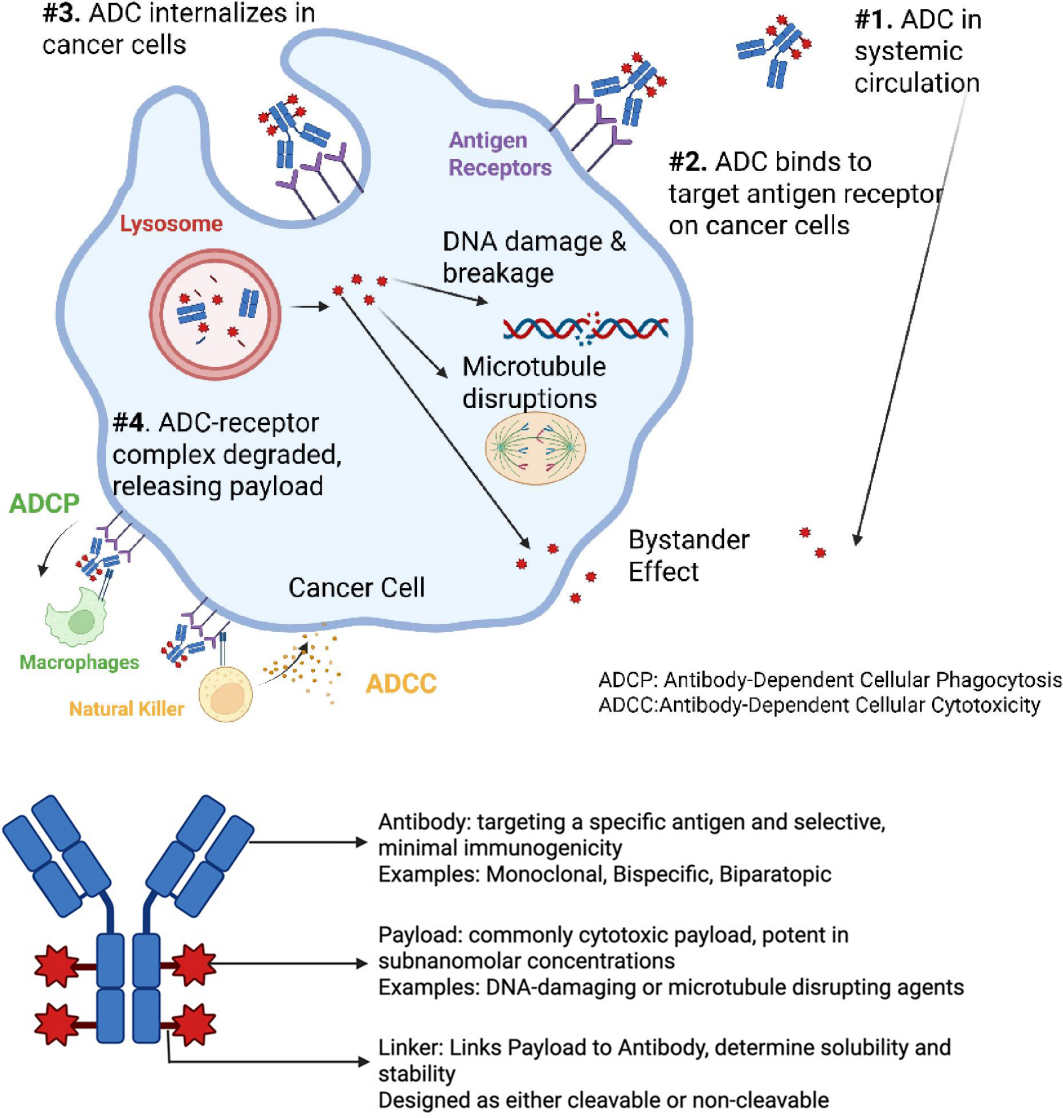
(ADC components and MOA) structure of an ADC, comprising an antibody that selectively binds a target antigen, a typically cytotoxic payload, and a linker that connects the antibody to the payload. General Mechanism, An ADC starts with the binding to a target antigen, undergoing an internalization into the cancer cell, leading to several different mechanisms of actions dependent on the payload including DNA damage/breakage, microtubule disruptions, and bystander effects. Created in BioRender. Lee, M. (2026) https://BioRender.com/g03c0ih and https://BioRender.com/mfo6wd7.

ADCs have developed into one of the most diverse and fastest growing classes of therapies in oncology and should not be viewed as a uniform class or merely as “targeted chemotherapy”. Their clinical behavior and outcomes reflect the complex interaction between target biology, antibody binding and internalization, linker stability, payload class, and host/tumor microenvironmental context. Unlike conventional targeted therapies such as small-molecule TKIs, which depend on suppressing an oncogenic signaling dependency, ADCs can exploit cell-surface antigens that may not themselves be oncogenic drivers, using the antibody as a delivery vehicle for potent cytotoxic or other functional payloads. Thus, ADCs represent a conceptual departure and have altered cancer therapy by separating tumor recognition from an effector mechanism, which differs from both conventional chemotherapy and traditional targeted therapy by physically coupling tumor-selective antigen recognition with ultra-potent intracellular cytotoxicity along with additional features such as bystander killing in which a payload can kill neighboring antigen-negative cells, immune effector engagement, or microenvironmental activation. This part of the review delves into the specific components and mechanisms of action for ADCs and how they function more like platform technology whose therapeutic behavior is determined by the interaction between target biology, internalization kinetics, linker chemistry, payload class, bystander effect, and host immune environment (**Figure 1**).

### 2.1 Design and mechanism of action of ADCs

By combining the advantages of targeted therapies and cytotoxic chemotherapy, this novel form of precision medicine enables more selective delivery of treatment to cancer cells, thereby improving the therapeutic index compared with conventional cytotoxic chemotherapy. The essential components of an ADC comprise an antibody that recognizes a tumor-associated antigen, a payload, and a linker, each with their own unique characteristics influencing the therapeutic index of ADCs and efficacy. However, these components do not function alone; each are required in a complex interplay that begins with selective binding of the monoclonal antibody component to a tumor-associated antigen/target (Fab) or receptor (Fc), followed by internalization of the ADC–antigen complex through receptor-mediated endocytosis (1). Once inside the cell, the traffic conjugates to endosomes and lysosomes, leading to the release of the active payload. This mechanism enables selective destruction of cancer cells while minimizing damage to normal tissues. An additional mechanism relates to a “bystander effect”. This refers to if a payload damages other nearby cells that were not the intended target, which may occur if payload diffuses out of the target cell to surrounding cells based on permeability and tumor microenvironment (1, 2). This understudied phenomenon highlights both the potential benefit of overcoming tumor heterogeneity and low-antigen expression but also off-target toxicity; this will be later examined in the review via various targets, providing a crucial viewpoint on the balance of efficacy and adverse effects (2–4). Lastly, immune-mediated mechanisms such as antibody-dependent cellular cytotoxicity (ADCC), antibody-dependent cellular phagocytosis (ADCP), Fc activation, and complement activation/recruitment of cytotoxic T cells make ADCs a multi-layered therapeutic platform that integrates precision targeting and moves beyond cytotoxic mechanisms to introduce its potential as a rational synergistic choice in combination with immunotherapy for lung cancer (1, 5).

#### 2.1.1 Antibody

The antibody component in the ADC serves as the targeting moiety. Most clinical ADCs use fully human or humanized IgG1 antibodies, which offer extended half-life and can engage Fc-mediated effector functions such as ADCC or phagocytosis and lower immunogenicity potential (6, 7). Ideally, the antibody should have a high binding affinity and be able to internalize into the tumor cells while also targeting antigens that are highly expressed on cancer cells but minimally on normal tissues (8, 9) However, Fc activity can also contribute to inflammatory or hepatic toxicities. Novel approaches to the antibody component of ADCs include Fc-silenced antibodies and aglycosylated monoclonal antibodies to minimize liver and inflammatory/immune toxicities (2, 10), incorporating multiple antibodies (bi-specific or tri-specific) that target more than one target. Antibody pro-bodies that are activated by pH changes to increase specificity and other post-translational modifications are currently being explored (2, 11).

#### 2.1.2 Payload

The payloads of ADCs are typically highly potent cytotoxins that tend to be more toxic than conventional chemotherapeutic agents (12, 13). Some of the most common payloads include topoisomerase I inhibitors (e.g. DXd, SN-38), microtubule inhibitors (e.g. MAF, MMAE), maytansinoids (e.g. DM1, DM4), calicheamicins, and PBDs (1, 14, 15). An important aspect of these payloads is their permeability and ability to diffuse across the membranes. This is dependent on payload hydrophobicity, membrane permeability, diffusion distance of the released free payload, tissue penetration, and characteristics of the tumor microenvironment (1, 16, 17). However, this effect can also lead to negative consequences, such as an increased ability to be cleared from circulation along with an increased ability to be immunogenic and cause off-target toxicities such as hepatotoxicity and ocular side effects (11, 15, 18). Unique class toxicities have been seen with MMAE causing neuropathy, DM1 with hepatoxicity, and thrombocytopenia and DM4/MMAF with ocular toxicity. Also, another key factor of ADCs is the drug-antibody ratio (DAR). There is no known ideal DAR; it is theorized that if the DAR is too low it could lead to underdosing whereas if the DAR is too high it can be cleared faster and cause more toxicity. Thus, balancing the DAR in ADCs has become a critical aspect to balance efficacy and toxicity. Newer approaches involving glycation with polyethylene glycol (PEG) aim to enable high-DAR ADCs (19) and reduce the side effect profiles of these agents (20, 21).

Other cytotoxic payloads that are being examined, such as tubulysins (anti-mitotic) (22, 23), duocarmycins (DNA alkylators) (24), PNU-159682 (a topoisomerase II inhibitor) (25), and amanitin (RNA polymerase II inhibitors) (26, 27), along with novel payloads such as immunomodulators, DACs (28), ISAC (29), and radioisotopes (e.g (30).Lu) (31, 32), seek to improve on these cytotoxic payloads (15).

#### 2.1.3 Linker

The last component of an ADC is the linker that combines the antibody with the payload. It functions to keep the ADC stable in circulation and prevent premature systemic toxicity while also releasing the payload efficiently in the tumor cell and tumor microenvironment (33). There are two types of linkers distinguishable on whether they are cleavable or non-cleavable. Non-cleavable linkers are stable bonds that resist proteolytic degradation but depend on endocytosis of the antibody and lysosomal degradation, thus ensuring stability in the systemic circulation and with the potential to reduce bystander effect and off-target diffusion (15). In contrast, cleavable linkers, which have become the preferred form of linkers, are hydrolysable and activated by tumor-associated factors (pH dependent), intracellular proteases glutathione reduction, lysosomal proteases, cathespins to maximize the ADC potency, and bystander effect (15, 34). However, this also raises the concern of a premature release of the payload prior to binding to the target and the stability of the linker has become critical. Ongoing research has started to focus on increasing the stability of these cleavable linkers through various release methods involving beta-glucuronidase (35), phosphatase (3), legumain (36), becoming responsive to cathepsin (37, 38), utilizing GGFG tetrapeptide linkers (15, 33, 39), along with also including hydrophilic groups to increase solubility (19).

## 3 Antibody target-driven landscape in lung cancer

### 3.1 Currently approved targets

#### 3.1.1 ERBB2 (HER2)

The HER2 is a transmembrane receptor tyrosine kinase encoded by the ERBB2 gene on chromosome 17q12, belonging to the epidermal growth factor receptor (EGFR/ErbB) family (40). *ERBB2*/*HER2* alterations—including overexpression, gene amplification, and activating mutations—are oncogenic drivers across multiple cancer types and represent one of the most successfully targeted molecular abnormalities in oncology (41). In approximately 2-5% of NSCLC, *HER2* mutations are present, with amplifications in around 2.0%, and protein overexpression ranging from 2 to 35%, each representing distinct molecular entities with different clinical implications and treatment responses (42, 43).

One of the earliest ADCs approved for NSCLC was trastuzumab deruxtecan (T-DXd) at a dose of 5.4mg/kg. T-DXd is a *HER2*-directed ADC that deliver a camptothecin-derived topoisomerase I inhibitor payload (DXd) via a cleavable linker and has a DAR of 8 (44).

In the phase II open-label single arm DESTINY-Lung 01trial (NCT03505710) (45) evaluating T-DXd at 6.4mg/kg, the cohort included *HER2*-mutants that were predominantly in exon 20 (86%) and exon 19 (7%) in the activating tyrosine kinase domain with additional mutations in exons 8 (7%, extracellular domain). The phase II single arm DESTINY-Lung 02 trial (NCT04644237) (46) also evaluated T-DXd at 5.4mg/kg or 6.4mg/kg in patients with *HER2*-mutant NSCLC; the majority were exon 20 alterations (93.4%, specifically mainly insertions and commonly p. Tyr772_Ala775dup), exon 19 (3.9%), exon 8 (2.0%), and exon 21 (0.7%, kinase domain).

Both trials also included patients with *HER2* amplification but in a smaller subset. In the DESTINY-Lung01 trial (45), half the cohort had no testing for HER2 protein expression and gene-amplification status, and it was evaluated in only 53 and 45 patients, respectively. Of the patients evaluated for HER2 protein expression, 83% (44/53) had an immunohistochemical score (IHC) of 1+ to 3+ with majority with IHC 2 +. Only 0.4% (2/45) had *HER2* gene amplification. In the DESTINY-Lung02 trial (46), *HER2* amplification was based on copy number gain but was not evaluated in the T-DXd 6.4mg/kg cohort. In the 5.4mg/kg cohort, a total of 63 of the total 102 patients were evaluated for *HER2* amplification with only 4.8% (3/63) were positive. Overall, although both DESTINY-Lung01 and DESTINY-Lung02 were highly encouraging, they were not powered to compare outcomes across distinct *HER2* alteration subgroups and, since amplification/protein-expression data were incomplete in substantial subsets, conclusions about broader HER2 biology beyond canonical *HER2*-mutant disease were limited.

Specifically, as for the clinical results, both the DESTINY-Lung 01trial and DESTINY-Lung02 trials demonstrate the clinical efficacy of T-DXd in previously treated *HER2* mutant NSCLC. The overall cohort in the DESTINY-Lung 01trial (45) had an objective response rate (ORR) of 55% (95% confidence interval [CI], 44 to 65), median progression-free survival (PFS) of 8.2 months (95% CI, 6.0 to 11.9), and median overall survival (OS) of 17.8 months (95% CI, 13.8 to 22.1). Similarly, in the DESTINY-Lung02 trial (46), ORR was 49% (95% CI, 39 to 59.1) and 56% (95% CI, 41.3 to 70), median PFS by blinded independent central review (BICR) of 9.9 months (95% CI, 7.4 to not evaluable [NE]) and 15.4 months (95% CI, 8.3, NE), and median OS of 19.5 months (95% CI, 13.6 to NE) and NE (95% CI, 12.1 to NE) respectively with T-DXd at 5.4mg/kg and 6.4mg/kg. A separate analysis that specifically evaluated patients with HER2 protein overexpression (IHC 3+/2+) without *HER2* mutations and who were treated with T-DXd at 6.4mg/kg (cohort 1) and 5.4mg/kg (cohort 1A) in DESTINY-Lung01 was examined. Clinical outcomes in cohort 1 and cohort 1A revealed an ORR of 26.5% (95% CI, 15.0, 41.1) and 34.1% (95% CI, 20.1, 50.6), median PFS of 5.7 months (95% CI, 2.8, 7.2) and 6.7 months (95% CI, 4.2, 8.4), and median OS of 12.4 months (95% CI, 7.8, 17.2) and 11.2 months (95% CI, 8.4, NE) respecitvely (47).

Moreover, in a pooled analysis of DESTINY-Lung01 and Lung02 in which both treated and untreated stable brain metastases were permitted at baseline, intracranial ORRs of 50% (7/14) and 30% (9/30), respectively, were observed at the 5.4mg/kg and 6.4mg/kg doses (48). Importantly, there are noted differences in responses between patients with different *HER2* alterations between the different exons or amplification statuses. However, these trials were not originally powered to evaluate these differences nor did every patient receive tumor testing for amplification, resulting in a small sample size of patients with data. Further research is needed to validate the use of T-DXd for broader use in patients with less common *HER2* mutants.

Lastly, in terms of safety, a significant toxicity of T-DXd is interstitial lung disease (ILD)/pneumonitis, which can be clinically significant and dose-dependent, requiring proactive monitoring, early recognition, and prompt intervention. In DESTINY-Lung02, adjudicated drug-related ILD/pneumonitis occurred in 12.9% of patients treated with 5.4 mg/kg versus 28.0% with 6.4 mg/kg, including fatal cases, and exposure–response analyses supported the lower dose as the preferred balance of efficacy and safety (46). Beyond ILD, common treatment-emergent adverse events include gastrointestinal and constitutional symptoms such as nausea, fatigue, vomiting, constipation, and alopecia, which are generally manageable but may accumulate over prolonged treatment and contribute to dose interruptions or reductions. Collectively, the safety profile underscores that, while T-DXd offers substantial efficacy, its optimal use depends on structured ILD surveillance pathways and disciplined management algorithms to minimize morbidity and prevent fatal pneumonitis.

Currently, T-DXd is approved only for previously treated patients with advanced HER2-mutant or HER2-overexpressing NSCLC, based on evidence from single-arm phase II trials. Confirmatory studies are ongoing, including DESTINY-Lung04 (NCT05048797), a randomized phase III trial comparing T-DXd with standard-of-care treatment in the first-line setting for patients with advanced HER2-mutant NSCLC (49).Combinations with immunotherapy and T-DXd have previously been evaluated in the phase Ib trial DESTINY-Lung03 trial (NCT04686305) (50), with T-DXd at 4.4 or 5.4mg/kg plus durvalumab and platinum-based chemotherapy in patients with HER2 over-expression (IHC 3+/2+). This trial evaluated three cohorts, with arm 1A with T-DXd, durvalumab, and cisplatin, arm 1B with T-DXd, durvalumab, and carboplatin, and arm 1D with T-DXd. Drug-related serious adverse events (SAEs) were reported in 63.6%, 37.5%, and 16.7% of patients, respectively, with ORR of 37.5% (arm 1B) and 44.5% (arm 1D). Overall, this trial did not support the use of the combination of T-DXd with durvalumab and platinum-based chemotherapy (50). There are still ongoing trials, such as the phase III trial DESTINY-Lung06 (NCT06899126) (51) with first-line T-DXd in combination with pembrolizumab compared to pembrolizumab with platinum-based chemotherapy in patients with HER2 over-expression (IHC 3+/2+) and PD-L1 TPS <50% and the phase II biomarker-directed umbrella HUDSON trial (NCT03334617) in patients previously treated with immunotherapy with multiple combination arms that includes T-DXd and is still awaiting results on the viability of this combination.

Recently, Trastuzumab rezetecan (SHR-A1811) has been positioned as a next-generation ADC directed towards *HER2* with a DAR of 6 and a highly permeable topoisomerase I inhibitor payload. Data on this ADC were released from the phase II HORIZON-Lung trial (NCT0481833) conducted in China (52). There was a reported ORR of 73% (95% CI 63.3 to 82.0), median PFS of 11.5 months (95% CI 9.9 to Not Reached (NR)), a safety profile of G3–4 of 62% with neutropenia (40%), decreased white blood cells (WBC) (27%), anemia (23%), thrombocytopenia (11%), and decreased lymphocytes (7%) (52). ILD was seen in 7% of patients with median time onset of 84 days. Treatment-related adverse events (TRAEs) led to dose interruption (30%), dose reduction (17%), and dose discontinuation (1%) (52). Further NSCLC trials of Trastuzumab rezetecan are ongoing in China in combination with Pertuzumab (NCT07377916) or Pyrotinib (NCT05482568).

#### 3.1.2 MET

MET (mesenchymal-epithelial transition) is a transmembrane receptor tyrosine kinase activated by its sole ligand, hepatocyte growth factor (HGF) (53, 54). Upon HGF binding, MET autophosphorylates at tyrosine residues Tyr1234 and Tyr1235 within the catalytic site, creating a multifunctional docking site that recruits adaptor proteins including GRB2, GAB1, and STAT3 (55). This activates downstream signaling through PI3K/AKT, RAS/RAF/MAPK, JAK/STAT, and Wnt/β-catenin pathways, regulating cell proliferation, survival, migration, motility, and angiogenesis (53, 56). MET can serve as an actionable oncogenic driver in NSCLC through exon 14 skipping mutations (2-4%), gene amplification (1-5% *de novo*; 10-25% as acquired resistance), and protein overexpression (>50% of tumor cells with IHC 3+) (19-25%) (57, 58).

Telisotuzumab vedotin (Teliso-V) is a c-MET-directed ADC comprising a monomethyl auristatin E (MMAE) payload linked to the antibody via a cleavable valine-citrulline dipeptide linker, with a DAR of 3. It received approval based on results from the pivotal single-arm phase II LUMINOSITY study (NCT03539536) (59, 60). Eligible patients included in this trial harbored both c-MET overexpression and were *EGFR*-wildtype and had prior treatments for advanced NSCLC. c-MET expression was defined as ≥25% tumor cells with 3+ staining as high (≥50%) and intermediate (≥25% to <50%). Although it was not the primary objective of the trial, Teliso-V did demonstrate a differential response based on c-MET expression. The ORR, median PFS, and OS in the c-MET high group was 34.6% (95% CI, 24.2 to 46.2), 5.5 months (95% CI, 4.1 to 8.3), 14.6 months (95% CI, 9.2 to 25.6) and 22.9% (95% CI, 14.4 to 33.4), 6.0 months (95% CI, 4.5 to 8.1), 14.2 months (95% CI, 9.6 to 16.6) in the c-MET intermediate cohort, respectively. In the overall cohort it was 28.6% (95% CI, 21.7 to 36.2), 5.7 months (95% CI, 4.6 to 6.9), and 14.5 months (95% CI, 9.6 to 16.6) respectively (60). The safety profile of Teliso-V reflects both ADC-associated and MMAE payload-associated toxicities, with peripheral neuropathy (30%) (G≥3 7%) and peripheral edema (16%) as the most characteristic adverse events and key cumulative dose-limiting concerns in LUMINOSITY trial (60). Other safety data reported was pneumonitis frequency of 10.5%, 56.4% experiencing G≥3 TRAEs, and 21.5% discontinuing due to TRAEs. Similar to T-DXd, since Teliso-V had undergone early accelerated approval based only on a single arm phase II trial, several confirmatory trials include a phase III trial, TeliMET NSCLC-01 study (NCT04928846), comparing Teliso-V versus docetaxel in previously treated patients with c-MET overexpression. Lastly, Teliso-V is also being examined in combination with osimertinib in a phase II trial (NCT07323641) for patients that have progressed after osimertinib. Both trials continue the biomarker-driven concept of targeting c-MET in both *EGFR* wildtype and mutant setting.

Another emerging MET-directed ADC is Telisotuzumab adizutecan (Temab-A). It contains a different topoisomerase 1 inhibitor payload with adizutecan and has a DAR of 6.2. In the Temab-A phase I trial (NCT05029882) with advanced solid tumors, reports of the subset of patients with *EGFR* mutant NSCLC with a median follow-up of 9.7 months were found to have a reported ORR of 63% (61) and also in *EGFR* wildtype NSCLC with ORR of 43.8% across all c-MET expression levels and clinical benefit rate of 85% (62, 63). In terms of safety, reported TRAEs >3 of 73% of patients, the majority of which were cytopenias (42-54%), nausea (42%), and ILD/pneumonitis of 6-7% (G≥3 2%) and discontinuation/reduction in 10% and 33% of patients (61, 62). Future trials on Temab-A include a phase II/III trial (NCT07155187) in patients with *EGFR*-mutant NSCLC who had previously been treated with EGFR TKI as well as combination trials, such as a phase II/III trial (NCT07005102) involving treatment naïve patients with *EGFR*-mutant metastatic NSCLC evaluating first-line Temab-A with osimertinib, and a phase I/II trial (NCT06772623) involving treatment naïve patients with no actionable alterations evaluating first-line Temab-A with immunotherapy (63).

#### 3.1.3 TROP2

TROP2 (trophoblast cell-surface antigen 2) is a type I transmembrane glycoprotein encoded by the *TACSTD2* gene, with limited expression in normal tissues but consistent overexpression across epithelial cancers (64, 65). It functions as an intracellular calcium signal transducer that promotes cell proliferation, migration, invasion, and metastasis through multiple oncogenic signaling pathways such as EGFR/AKT, ERK/MEK, FAK, NF-κB, and STAT (64–68). TROP2 protein expression (any level) is observed in 82-91% of NSCLC across sample sets, with high TROP2 expression (IHC 2+/3+) reported in 64-75% of adenocarcinomas and 75% of squamous cell carcinomas (69–71).

Datopotamab deruxtecan (Dato-DXd) is a TROP2-directed ADC linked with a tetrapeptide-based linker to a topoisomerase I inhibitor payload (DXd) with a DAR of 4. Although it was originally developed for use across broad NSCLC populations, Dato-DXd received accelerated approval in patients with *EGFR*-mutant NSCLC based on the TROPION-Lung01/05 results (72, 73). In the phase III TROPION-Lung-01 trial (NCT04656652) (72), patients with pretreated advanced or metastatic NSCLC were randomized to either Dato-DXd or docetaxel without any biomarker criteria required. The overall median PFS was significantly higher in the Dato-DXd cohort at 4.4 months (95% CI, 4.2 to 5.6) compared to docetaxel at 3.7 months (95% CI, 2.9 to 4.2) (HR 0.75) (95% CI: 0.62 to 0.91) (p=0.004). However, in the overall cohort, the OS was not significantly different between Dato-DXd compared to docetaxel at 12.9 months (95% CI, 11.0 to 13.9) versus 11.8 months (10.1, 12.8) (HR 0.94) (95% CI: 0.78 to 1.14) (p=0.530). With further subgroup analysis, patients with non-squamous histology had significantly higher PFS at 5.5 versus 3.6 months (HR 0.63) (95% CI: 0.51 to 0.79) but again OS not significantly different at 14.6 months versus 12.3 months (HR 0.84) (95% CI: 0.68 to 1.05) respectively with Dato-DXd and docetaxel. The single-arm phase II trial with TROPION-Lung05(NCT04484142) (73) involved patients with advanced/metastatic NSCLC harboring actionable alterations who had progressed on targeted therapy and platinum-based chemotherapy with Dato-DXd. The majority of these patients had *EGFR* alterations (n=78 patients, 56.9%) with the majority sensitizing or T790M mutations (80.9%). Other alterations included rearrangements in *ALK* (n=34 patients, 24.8%), *ROS1* (n=10 patients, 7.3%), *RET* (n=8 patients, 5.8%), *MET exon 14* skipping (n=5 patients, 3.6%). *BRAF* (n=4 patients, 2.9%), and *MET* amplification (n=3 patients, 2.2%). The overall cohort had an ORR of 35.8% (95% CI, 27.8 to 44.4), DCR of 78.8% (95% CI, 71 to 85.3), and median PFS 5.4 months (95% CI, 4.7 to 7.0) and median OS of 13.6 months (95% CI, 9.9 to NE) (73). The EGFR and ALK sub-cohorts were also analyzed with ORR of 43.6% (95% CI, 32.4 to 55.3) and 23.5% (95% CI, 10.7 to 41.2), DCR of 82.1% (95% CI, 71.7 to 89.8) and 73.5% (95% CI, 55.6 to 87.1), median PFS of 5.8 months (95% CI, 5.4 to 8.3) and 4.3 months (95% CI, 2.6 to 6.9), and lastly median OS 18.3 months (95% CI, 12.4 to NE) and 9.3 months (95% CI, 5.8 to 18.3) respectively (73) along with an intracranial ORR of 22% (95% CI, 6 to 48) (74). The clinical utility of Dato-DXd in the *EGFR-*mutant setting was further supplemented in a pooled analysis of both TROPION-Lung01 and Lung05 that focused on 117 patients (75). Similarly, the ORR in this combined cohort was 43% (95% CI, 34 to 52), with 4% of patients having a complete response (CR). The median PFS was 5.8 months (95% CI, 5.4 to 8.2) and median OS was 15.6 months (95% CI, 13.1 to 19.0) (75). The most frequent toxicity was stomatitis/mucositis ranging from 55-65% across all grades with grade >3 of 9-11% (72, 73, 75) in addition with ocular/ophthalmologic toxicities, cytopenias such as anemia, neutropenia, thrombocytopenia, and ILD/pneumonitis. Other common toxicities included nausea, ranging from 46-55%, and alopecia 50%, decreased appetite, fatigue, constipation, vomiting, rash, and asthenia, most of which was low-grade.

Multiple trials of Dato-DXd are currently ongoing. These include the phase III TROPION-Lung08 trial (NCT05215340), which is evaluating first-line treatment in patients with advanced NSCLC lacking actionable genomic alterations and with PD-L1 expression >50%, randomized to receive either Dato-DXd plus pembrolizumab or pembrolizumab alone (76). The phase III TROPION-Lung10 trial (NCT06357533) is similarly investigating first-line therapy, randomizing patients to Dato-DXd plus rilvegostomig or Dato-DXd alone (77). In patients with PD-L1 expression <50%, the phase III TROPION-Lung07 trial (NCT05555732) is evaluating Dato-DXd in combination with pembrolizumab and platinum-based chemotherapy versus pembrolizumab and platinum-based chemotherapy alone (78). Lastly, there is a phase III trial examining patients with non-squamous NSCLC without actionable alterations and who were previously treated with immunotherapy with TROP2 expression based on a novel biomarker assessment and a normalized membrane ratio (NMR) in TROPION-Lung17 (NCT07291037) that aims to confirm findings seen in TROPION-Lung01 in this patient population and further described in the review.

Sacituzumab govitecan is another TROP2-directed ADC that carries the topoisomerase I inhibitor SN-38 as its payload and has a DAR of 7.6. Although it is not currently FDA-approved for NSCLC, it remains under investigation for the treatment of both NSCLC and SCLC (79). In the initial phase I/II trial (NCT01631552), Sacituzumab govitecan showed potential promising durable responses (80). However, in the EVOKE-01 (NCT05089734) (81) trial, with patients who were previously treated for advanced NSCLC, OS was not significantly different between those treated with Sacituzumab govitecan or docetaxel, with 11.1 versus 9.8 months (HR 0.84) (95% CI 0.68 to 1.04) (p=0.0534). In this trial, TRAEs G≥3 were experienced in 66.6% vs 75.7% of patients, respectively. In the subsequent phase II EVOKE-02 trial (NCT05186974) (82), the combination of Sacituzumab govitecan with pembroluzimab was evaluated for patients with treatment-naive metastatic NSCLC with analysis based on TPS of either >50% (cohort A) or <50% (cohort B). For cohort A and B, the ORR was 66.7% (95% CI 47.2 to 82.7) and 29.0% (95% CI 18.2 to 41.9) and PFS of 13.1 months (95% CI 6.7 to NR) and 7.0 months (95% CI 4.2 to 12.9) respectively (82). This reveals that the combination was more effective in cohort A with TPS >50% and TRAEs G≥3 noted in 76.1% (82). An ongoing phase III EVOKE-03 trial (NCT05609968) is comparing first-line pembrolizumab with the combination of pembrolizumab with Sacituzumab govitecan in patients TPS >50% NSCLC to confirm EVOKE-02 trial results.

Sacituzumab tirumotecan (sac-TMT) is another TROP2-directed ADC comprised of a payload containing belotecan-derived topoisomerase I inhibitor with a DAR of 7.4; it has emerged with promising results from both phase I/II trials (83) and the phase III OptiTROP-Lung04 trial (NCT05870319) conducted in China (84). In this phase III trial, patients had *EGFR*-mutant advanced NSCLC after progressing on an EGFR-TKI and were randomly assigned to receive sac-TMT or platinum-based chemotherapy. With a median follow-up of 18.9 months, the reported prespecified final analysis of PFS was 8.3 months and 4.3 months (HR 0.49) (95% CI 0.39 to 0.62), OS (HR 0.49) (95% CI, 0.39 to 0.62) (p=0.001), and 18-month OS rate of 65.8% and 48.0% for sac-TMT and platinum-based chemotherapy cohorts respectively (84). In terms of safety, TRAEs of G≥3 occurred in 58.0% of patients receiving sac-TMT and in 53.8% of those receiving platinum-based chemotherapy respectively, with the most common being neutropenia (39.9% vs. 33.0%). It was granted an FDA breakthrough therapy designation in 2024 and is awaiting further confirmatory trials with phase III TroFuse-004 study (NCT06074588) that is being conducted in the United States along with a front-line trial in combination with tagitanlimab, an anti-PD-L1 with the phase II OptiTROP-Lung01 study (NCT05351788) (85).

SHR-A1921, another potential emerging TROP2 ADC, contains a digestible maleimide tetrapeptide (GGFG)-containing linker that can help improve its stability in plasma and a DNA topoisomerase I inhibitor payload (86). In a phase I trial (NCT05154604), which included 148 patients with NSCLC (of which 51.4% harbored actionable genomic alterations, aka AGAs) and 18 patients with SCLC along with various other advanced cancers, the overall TRAEs of G≥3 was 33.8% and 2.6%, respectively, with patients discontinuing treatment due to TRAEs, with ILD (0.8%) being the main cause. Efficacy data showed an ORR of 27.8% (95% CI, 9.7 to 53.5) for SCLC, 26.6% (95% CI, 17.3 to 37.7) for AGA-positive NSCLC, and 21.8% (95% CI, 13.2 to 32.6) for AGA-negative NSCLC, with a noted high incidence in this subgroup of G2/G3 stomatitis ranging 44.3% to 72.7%. Lastly, TROP-2 expression was examined based on H-score (low 0-100, medium 100-200, high >200 to 300) specifically in NSCLC, in which ORR was 20.5% (95% CI, 9.3 to 36.5), 32.0% (95% CI, 12.8 to 64.9) and 35.7% (95% CI, 12.8 to 64.9) and PFS of 4.6 months (95% CI, 1.4 to 12.7), 5.6 months (95% CI, 2.7 to 7.6) and 6.2 months (95% CI, 4.1 to 9.3) respectively.

Summary of the currently approved ADCs in **Table 1**.

**Table 1:** Summary of currently approved ADCs (trial data and toxicities).

ADCs/trials (phase)	Patient population	Follow-up (median)	ORR (%)	PFS (median)	OS (median)	Common toxicities (any grade/grade >3; %)
T-DXd — DESTINY-Lung01 (Phase II) (45) NCT03505710	Advanced NSCLC (HER2-mutant); previously treated; stable brain mets allowed	13.1 months	55%	8.2 months	17.8 months	96.7%/69.2% (overall) ILD/pneumonitis (20.9/4.4%) Neutropenia (35.2%/19%) Anemia (36.3%/10%) Nausea (75.8%/8.8%) Fatigue (60.4%/6.6%) Vomiting (46.2%/5.5%) Diarrhea (40.7%/37.4%)
T-DXd — DESTINY-Lung02 (Phase II; 5.4 mg/kg arm) (46) NCT04644237	Advanced NSCLC (HER2-mutant); previously treated; stable brain mets allowed	15.8 months	49%	9.9 months	NR (interim)	96.0%/38.6% (overall) ILD/pneumonitis (12.9%/1.0%)
Teliso-V — LUMINOSITY (Phase II) (60) NCT03539536	Advanced NSCLC (Non-squamous EGFR-wildtype with c-MET overexpression; previously treated	11.2 months	28.6% (34.6% MET- high 22.9% intermediate)	5.7 months	14.5 months	97.1%/56.4% (overall) ILD/pneumonitis (10.5%) Peripheral sensory neuropathy (30.2%/7.0%) Peripheral edema (16.3%/1.7%) fatigue (14.0%/2.3%) Decreased appetite (11.6%/0.6%) Increased ALT (11.0%/3.5%) Hypoalbuminemia (10.5%/0%)
Dato-DXd — TROPION-Lung05 (Phase II) (73) NCT04484142	Advanced NSCLC (actionable alterations including EGFR and ALK); previously treated with targeted therapy	14.8 months	35.8% (44% EGFR-mut 24% ALK)	5.4 months	13.6 months	94.2%/27.7% (overall) ILD/pneumonitis (3.6%/0.7%) Stomatitis/mucositis (56.2.10.9%) Ocular surface events (26.3%/2.2%) Infusion-related reactions (16.1%/0%) Nausea (54.7%/2.2%)
Dato-DXd — TROPION-Lung01 (Phase III) (72) NCT04656652	Advanced NSCLC; previously treated; Dato-DXd versus Docetaxel	10.9 months	26.4% versus 12.8% (all) NSQ 31.2% versus 12.8% SCC 9.2% versus 12.7%	4.4 versus 3.7 months (all) NSQ 5.5 versus 3.6 months SCC 2.8 versus 3.9 months	12.9 versus 11.8 months (all) NSQ 14.6 versus 12.3 months SCC 7.6 versus 9.4 months	Dato-DXd: 87.5%/25.6% (overall) ILD/pneumonitis (8.8%/3.7%) Stomatitis (47.5%/6.7%) Nausea (34%/2.4%) Diarrhea (10.1%/0.3%) Anemia (14.8%/4.0%)

NSQ, Non-squamous NSCLC; SCC, squamous NSCLC.

### 3.2 Emerging potential targets

Newer targets have been explored to broaden and further expand treatment options. Although it is beyond the scope of this manuscript, we would like to acknowledge that the field of SCLCs has experienced multiple rapid developments due to the use of ADCs, with notable examples including B7-H3 [Ifinatamab deruxtecan (87), YL201 (88, 89)], SEZ6 [ABBV-706 (90)], DLL3 [Zocilurtatug pelitecan (91), SHR-4849/IDE849 (92)], and TROP2 [Sacituzumab govitecan (93)]. The emerging targets and antibody type of treatments included below have shown promising preliminary results, although it remains to be seen how they will fit into the future paradigms of management and in this section of the review these targets have reported clinical trial data but have no currently approved ADCs yet.

#### 3.2.1 ERBB3 (HER3)

ERBB (HER3) is a member of the ERBB/HER receptor tyrosine kinase family that lacks intrinsic kinase activity but functions as a critical signaling hub through heterodimerization with other ERBB family members, particularly HER2 and EGFR. It is activated by binding to its ligands neuregulin-1 (NRG1) and neuregulin-2 (NRG2), with six binding sites for the p85 regulatory subunit of phosphoinositide 3-kinase (PI3K), making it an activator of the PI3K/AKT/mTOR pathway, regulating cell proliferation, survival, differentiation, metabolism, and migration (94, 95). In normal tissues, HER3 expression is limited, but it becomes overexpressed in 83% of NSCLC (96), and up to 85-100% of patients harbor an *EGFR* alteration; it has also been associated with resistance to EGFR directed therapy (97–100) and is detectable in 91% of brain metastases (96).

Patritumab deruxtecan (HER3-DXd) is a HER3-directed ADC comprising a topoisomerase I inhibitor payload via a cleavable tetrapeptide linker; it has a DAR of 8 and has been evaluated in patients with *EGFR* mutant NSCLC. In the single-arm phase II HERTHENA-Lung01 trial (NCT04619004) on HER3-DXd in patients with *EGFR*-mutant who were previously treated with EGFR TKIs, promising results were shown, with ORR by BICR of 29.8% (95% CI, 23.9, 36.2), median PFS of 5.5 months, median OS of 11.9 months, and CNS ORR of 33.3% (95% CI, 17.3, 52.8) (101). In terms of safety, 64.9% had G≥3 TRAEs with most commonly hematologic toxicities such as thrombocytopenia (20.9%) and neutropenia (19.1%). However, in the confirmatory phase III HERTHENA-Lung02 trial (NCT05338970) (102) that compared HER3-DXd versus platinum-based chemotherapy, it was revealed that, although there was PFS benefit 5.8 months versus 5.4 months (HR 0.77) (95% CI, 0.63 to 0.94) (p=0.011), it did not meet its OS endpoint with 16.0 months versus 15.9 months (HR 0.98) (95% CI 0.79 to 1.22). Based on these results, the FDA biologics license application (BLA) has since been voluntarily withdrawn, but ongoing studies such as the phase I/II KEYMAKER-U01 substudy 01G (NCT06731907) is evaluating HER3-DXd in combination with pembrolizumab with/without platinum chemotherapy in treatment naïve patients and solid tumors harboring NRG1 fusion (NCT06383884).

Other HER3 ADCs that are in development include DB-1310, which also contains a topoisomerase I payload and DAR of 8 and has shown early preliminary efficacy in a phase I/II trial (NCT05785741), with ORR of 35.7% (95% CI, 21.55 to 51.97) in patients with *EGFR*-mutant NSCLC, median PFS of 7.0 months, and G>3 TRAEs in 30.9% of patients. The most common TRAEs include nausea, anemia, neutropenia, and thrombocytopenia (103–105). Another HER3 ADC in early development is AMT-652, conjugated to exatecan (which has higher cytotoxic potency than DXd) via a modified self-immolative PABC spacer (106). Preclinical studies showed potent antitumor responses in low HER3 expression models, along with synergistic efficacy in combination therapies in cynomolgus monkeys (106). Otherwise, it remains to be seen if HER3 can still be a promising target for patients with *EGFR*-mutant NSCLC.

#### 3.2.2 CEACAM5

Carcinoembryonic antigen-related cell adhesion molecule 5 (CEACAM5) is a member of the CEACAM family, which comprises 12 different human proteins located on chromosome 19q13.2 (107). In normal adult tissues, CEACAM5 has limited expression, primarily found on the apical surface of epithelial cells in the gastrointestinal tract (108). The protein participates in multiple biological processes including cell-cell adhesion, intercellular signaling, cell differentiation, and immune response modulation and serves as a receptor for certain pathogens (108–111). In cancer, CEACAM5 promotes cell proliferation, migration, and malignant transformation through the activation of signaling pathways including p38-Smad2/3 expression, which is regulated by transcription factors including WTAP (Wilms’ tumor 1-associating protein), which promotes NSCLC progression through CEACAM5 upregulation (112–114). Its expression is particularly elevated in carcinomas of the gastrointestinal tract (colorectal, gastric, and pancreatic), genitourinary system, respiratory system, and breast (108, 115). CEACAM5 high expression (defined as ≥2+ intensity in ≥50% of tumor cells by IHC) occurs in approximately 20-24% of non-squamous NSCLC cases (116, 117).

Tusamitamab ravtansine (SAR408701) is a CEACAM5 ADC that is conjugated to the payload ravtansine (DM4, a maytansinoid microtubule inhibitor) with a DAR of 3.8; it had initially shown promising activity in CEACAM5-high non-squamous NSCLC with ORR of 20.3% (95% CI, 12.3% to 31.7%) in CEACAM5-high expressing tumors versus 7.1% (95% CI, 2% to 22.7%) in moderate expression (as ≥2+ intensity in 1-49% of tumor cells by IHC) (118) along with ORR of 41.7% in patients with high circulating CEA (≥100 µg/L) versus 8.1% in low CEA (<100 µg/L) patients (119). However, despite these initial results, it did not translate to durable clinical outcomes during the prespecified interim analysis of the phase III CARMEN-LC03 trial (NCT04154956) (120). The trial compared Tusamitamab ravtansine to docetaxel in patients with advanced non-squamous NSCLC previously treated with high CECAM5 positivity by IHC. The results from this trial revealed that both the OS and PFS for the Tusamitamab ravtansine cohort, 12.8 months (95%CI, 11.8 to 14.2) and 5.4 months (95% CI, 3.7 to 7.0) respectively, were not significantly different from docetaxel cohort of 11.5 months (8.9 to 15.2) and 5.9 months (95% CI, 5.5 to 7.4) respectively (120) and thus Sanofi has ended its development.

Other CEACAM5-targeted ADCs are in development, including precemtabart tocentecan (M9140), which carries the topoisomerase I inhibitor exatecan as its payload and has a DAR of 8. It has shown encouraging early clinical activity in metastatic colorectal cancer (121), with expansion cohorts in the phase Ib/II PROCEADE PanTumor trial (NCT06710132) ongoing in NSCLC and gastrointestinal malignancies, including colorectal, gastric, and pancreatic cancers (122). Another agent, BG-C477, also incorporates a topoisomerase I inhibitor payload with a DAR of 8 and has demonstrated initial preclinical activity, with a phase I trial currently underway (NCT06596473) (123).

#### 3.2.3 ITGB6

Integrin β6 (ITGB6) is a cell-surface adhesion receptor that forms the epithelial-specific αvβ6 heterodimer with integrin αv and is upregulated across many carcinomas, including NSCLC (reported as high as 50-90%) (124–126), where it frequently co-expresses with EGFR but has not been linked to prognosis or clinical outcomes (127). Sigvotatug vedotin (SGN-B6A) is an ITGB6 ADC conjugated to an MMAE vedotin payload with a DAR of 4, with phase I trial (NCT04389632) (128) results showing an ORR of 19.5% (95% CI, 12.6 to 28.0) and median PFS of 3.5 months (95% CI, 2.7 to 4.9) in all NSCLC patients and a higher ORR of 32.5% (95% CI, 18.6 to 49.1) and median PFS of 6.4 months (95% CI, 4.9 to 10.5) in taxane-naïve non-squamous NSCLC. Toxicity with G ≥3 TRAEs is detectable in 46% of patients, most commonly with dyspnea (9.7%), fatigue (7.1%), and neutropenia (5.3%) and treatment discontinuation in 13.3% (128). Currently, in order to confirm these findings, two phase III trials are underway with Be6A Lung-01(NCT06012435) (129), in which Sigvotatug vedotin is compared to docetaxel in previously treated advanced NSCLC and Be6A Lung-02 patients (NCT06758401) (130) in which Sigvotatug vedotin is combined with pembrolizumab compared to pembrolizumab PD-L1 high (≥50% TPS) advanced NSCLC in the first-line setting.

#### 3.2.4 Nectin-4

Nectin-4 is a calcium-independent immunoglobulin-like cell–cell adhesion molecule that is minimally expressed in healthy adult tissues but upregulated in embryonic and placental tissue; it is also implicated in several malignancies, with reports of overexpression in over 60% of NSCLC tumors (131–133). The most developed Nectin-4 ADC is Enfortumab vedotin (EV), an MMAE-based ADC that has become standard of care in metastatic urothelial carcinoma and is now being evaluated in patients who were previously treated with NSCLC in the phase II trial EV-202 (NCT04225117) (134). This trial enrolled 23 squamous and 43 non-squamous lung cancer patients but unfortunately the ORR, median PFS, and OS was 4.3% (95% CI, 0.1 to 22.0%) and 14.0% (95% CI, 5.3 to 27.9); 3.5 months (95% CI, 1.9, 5.4) and 4.1 months (95% CI, 2.8, 5.7); and 8.2 months (95% CI, 5.5 to 10.4) and 10.5 months (95% CI, 8.1 to 13.1) respectively. The most common TRAEs noted in the non-squamous cohort were rash maculopapular and peripheral sensory neuropathy (37.2% each); pruritus (34.9%); and alopecia (32.6%). There are several next-generation Nectin-4 ADCs also in development, including ADRX-0706, which delivers the tubulin inhibitor AP052 via a cleavable linker with DAR 8 and is currently in phase 1a/b testing (NCT06036121) with noted responses across different tumor types including NSCLC (135). There is also BAT8007, which uniquely employs an exatecan topoisomerase I payload rather than a tubulin agent, with preliminary phase I data (NCT05879627) showing tolerability and a subject with NSCLC achieving a partial response (136) and CRB-701 (SYS6002) with noted responses and tolerability that included NSCLC patients (NCT06265727) (137). It remains to be seen how Nectin-4 ADCs may fit into NSCLC treatments, especially given their responses noted in squamous cohorts.

#### 3.2.5 PTK7

Protein Tyrosine Kinase 7 (PTK7) is a receptor tyrosine kinase family pseudokinase that lacks catalytic activity but can modulate tumorigenesis and angiogenesis through multimerization with other cell-surface proteins and interaction with WNT signaling. It is upregulated in many solid and hematologic malignancies, including lung adenocarcinoma, with reported overexpression in 11-47% of NSCLC tumors (138, 139). Another unique finding was that there is potentially complementary expression with MET in patients with non-squamous *EGFR* wildtype NSCLC (140). Around 32% of patients were positive for MET overexpression, PTK7 expression, or both (140) but clinical development has been challenging. The first PTK7-directed ADC evaluated, Cofetuzumab pelidotin, which has a payload that was auristatin-based, demonstrated acceptable toxicity in the phase I dose-escalation trial (NCT02222922) (141) and in phase Ib trial (NCT04189614) (142). Responses in patients with previously treated EGFR–wild-type non-squamous NSCLC and high PTK7 expression (≥90%/≥2+) included an ORR of 21.4% (95%, 27.5 to 66.1) and 18.0% (95% CI, 28.6 to 54.3) compared to the overall study population. Despite these signals, further development was discontinued in August 2023 during phase 1 testing. MTX-13 seeks to improve on PTK7 ADC with a novel antibody (Ab13) with improved binding properties and exatecan payload (topoisomerase I inhibitor) instead of auristatin, novel self-immolative moiety (T1000), and a DAR of 8 with early preclinical data support (143). Other pending PTK7 ADCs in development that would benefit lung cancer patients are LY4175408 (NCT07046923) and DAY301 (NCT06752681), which are being explored as potential chimeric antigen receptor-modified T cells (CAR T) targets for lung cancer (144).

#### 3.2.6 AXL

AXL is a transmembrane receptor tyrosine kinase that promotes cancer growth and metastasis through signaling pathways such as PI3K/Akt and MAPK/ERK and is frequently overexpressed or amplified in epithelial malignancies including NSCLC. It is also implicated in resistance to targeted therapies like EGFR-TKIs, making it an appealing therapeutic target for ADCs. Several AXL-directed ADCs have entered development, though early candidates faced setbacks: Mipasetamab uzoptirine, a PBD-dimer ADC with a DAR of ~2, showed significant grade ≥3 toxicity leading to discontinuation in 2020 (145), while Enapotamab vedotin, an MMAE-based ADC, was also halted after failing to meet efficacy benchmarks alongside substantial toxicity (146). In contrast, Mecbotamab vedotin (CAB-AXL-ADC), a pH-sensitive ADC designed for selective tumor microenvironment binding and carrying an MMAE payload with DAR of 4, has demonstrated promising interim phase II trial (NCT04681131) activity in heavily pretreated non-squamous NSCLC either monotherapy or combined with nivolumab, with an ORR of 27.8% (95% CI, 9.7 to 53.5) in EGFR–wild-type patients post–PD-1 therapy and a safety profile that includes liver function test elevations, neutropenia, and peripheral neuropathy (147). Overall, further studies and results are required to confirm whether AXL is a promising target or not.

#### 3.2.7 MUC1

Mucin1 (MUC1) is a glycosylated transmembrane mucin family membrane protein that normally acts as a physical barrier between normal epithelial tissues and. MUC1 overexpression is seen in various cancers including breast, bladder and lung cancer (148) and leads to the activation of signaling pathways such as PI3K, MAPK, NFκB and Wnt/β, resulting in tumor migration, invasion, and accelerated growth with measurable subunits such as antigen CA 27.29 and CA 15-3 (149). Sacomitatug deruxtecan (DS-3939a) is an ADC composed of anti-TA-MUC1 antibody, peptide-based cleavable linker, and DXd as its payload and was shown to have preclinical *in-vitro* and *in-vivo* responses (148), Furthermore, it is being evaluated in a phase I/II trial (NCT05875168) (150) in patients with advanced tumors that included NSCLC; initial preliminary results revealed toxicities of nausea (57.4%), vomiting (36.2%), anemia (23.4%), and constipation (27.7%). Grade 3 or higher TRAEs occurred in 40.4% of patients; the most common (>2%) included decreased neutrophil count (15.6%), anemia (10.9%), and decreased platelet count (3.1%). All-grade adjudicated treatment-related ILD/pneumonitis occurred in 12.8% of patients. (151)Preliminary efficacy results revealed four patients with partial response and 11 with stable disease out of the 16 NSCLC patients (151).

### 3.3 Early Targets

The targets discussed in this section include early evaluations, with further results pending from phase I/II trials assessing the safety and efficacy of ADCs directed against these targets. A summary of the emerging and early targets can be found in **Table 2**.

**Table 2:** Emerging targets and ADCs in NSCLC.

Targets	ADCs	Trials (phase)	ORR (%)	PFS (median)	OS (median)	Common toxicities (any grade/grade >3; %)
ERBB (HER3)	Patritumab deruxtecan (HER3-DXd)	HERTHENA-Lung01 trial (Phase II) (101) NCT04619004	29.8%	5.5 months	11.9 months	99.6%/64.9% (5.6mg/kg) (overall) ILD (5.3%)* Thrombocytopenia (43.6%/20.9%) Neutropenia (26.2%/19.1%) Anemia (33.3/14%) Nausea (65.8%/3%) Decreased appetite (42.2%/3%) Constipation (34.2%/0%)
	Patritumab deruxtecan versus platinum chemotherapy	HERTHENA-Lung02 trial (Phase III) (102) NCT05338970	35.2% (HER3-DXd) 25.3% (platinum-chemotherapy)	5.8 months (HER3-DXd) 5.4 months (platinum-chemotherapy)	16.0 months (HER3-DXd) 15.9 months (platinum-chemotherapy)	100%/73% (HER3-DXd) (overall) ILD (5%)* Thrombocytopenia (52.1%/30%) Nausea (57.9%)* Fatigue (50.3%)*
	DB-1310	NCT05785741 (Phase I/II) (103)	35.7%	7.0 months	N/A	30.9% (G3) ILD (5.7%)* Nausea (36.6%/0.8%) Anemia (35.8%/4.1%) Neutropenia (34.1%/17.9%) Thrombocytopenia (31.7%/9.8%)
	AMT-562	NCT06199908 (Phase I)	N/A	N/A	N/A	N/A
CEACAM5	Tusamitamab ravtansine (SAR408701) versus Docetaxel	CARMEN-LC03 trial (Phase III) (120) NCT04154956	21.7%	5.4 months	12.8 months	95.9%/40.7% (overall)
	Precemtabart tocentecan (M9140)	PROCEADE PanTumor (Phase Ib/II) NCT06710132 (122)	N/A	N/A	N/A	N/A
	BG-C477	NCT06596473 (Phase I) (123)	N/A	N/A	N/A	N/A
ITGB6	Sigvotatug vedotin (SGN-B6A)	NCT04389632 (Phase I) (128)	19.5% (NSCLC) 32.5% (NSQ)	3.5 months (NSCLC) 6.4 months (NSQ)	N/A	G>3 46% (overall) Dyspnea (9.7%) Fatigue (7.1%) Neutropenia (5.3%)
	Sigvotatug vedotin versus Docetaxel	Be6A Lung-01 (Phase III) NCT06012435 (129)	N/A	N/A	N/A	N/A
	Sigvotatug vedotin + Pembrolizumab versus Pembrolizumab	Be6A Lung-02 (Phase III) NCT06758401 (130)	N/A	N/A	N/A	N/A
Nectin-4	Enfortumab vedotin	EV-202 (Phase II) NCT04225117 (134)	4.3% (SCC) 14.0% (NSQ)	3.5 months (SCC) 4.5 months (NSQ)	8.2 months (SCC) 10.5 months (NSQ)	Overall 82.6%/43.5% (SCC) 90.7%/30.2% (NSQ) Rash 21.7%/4.3% (SCC) 37.2%/7.0% (NSQ) Peripheral sensory neuropathy 21.7%/0% (SCC) 37.2%/2.3% (NSQ)
	ADRX-0706	NCT06036121 (Phase IA/B) (135)	N/A	N/A	N/A	N/A
	BAT8007	NCT0587962 (Phase I) (136)	N/A	N/A	N/A	N/A
PTK7	Cofetuzumab pelidotin	NCT04189614 (Phase Ib) (142)	18.0% (overall)	5.5 months (overall)	12.6 months (overall)	100%/69% (overall) Alopecia (52%/0%) Neutropenia (43%/37%) Pruritus (35%/2%) Headache (34%/3%)
AXL	Mecbotamab vedotin (BA3011) with Nivolumab	NCT04681131 (Phase II) (147)	27.8% (overall)	N/A	N/A	Liver function test elevations (N/A) Neutropenia (N/A) Peripheral neuropathy (N/A)
NaPi2b	TUB-040	NAPISTAR 1-01 (Phase I/II) NCT06303505 (152)	N/A	N/A	N/A	N/A
Folate Receptor Alpha	LY4170156	NCT06400472	N/A	N/A	N/A	N/A
	ZW191	NCT06555744	N/A	N/A	N/A	N/A
ROR2	Ozuriftamab vedotin (BA3021) with Nivolumab	NCT03504488	N/A	N/A	N/A	N/A
MUC1	Sacomitatug deruxtecan					

*any grade adverse events.

N/A, not available.

NSQ, Non-squamous NSCLC.

#### 3.3.1 NaPi2b

NaPi2b is a sodium-dependent phosphate transport protein frequently overexpressed in cancer, particularly in NSCLC adenocarcinoma, where retrospective surgical series have shown NaPi2b-high expression in up to 66% of adenocarcinomas compared with minimal expression in squamous tumors (153). Despite its appeal as a biomarker, development of NaPi2b-targeted ADCs has been challenging: Lifastuzumab vedotin, an MMAE-based ADC, demonstrated limited activity in NSCLC (8% ORR) and was discontinued after failing to improve PFS in platinum-resistant ovarian cancer (154, 155), while Upifitamab rilsodotin, an auristatin ADC with very high DAR (12–15), was canceled in 2023 after phase 2 failure to meet its ORR endpoint and serious safety concerns including fatal bleeding events (156–158). More recently, TUB-040, an Fc-silenced monoclonal antibody targeting NaPi2b with an exatecan-based NaPi2b ADC with DAR 8, has shown promising preclinical stability, potency, and tolerability in primate models with an ongoing phase I/II trial NAPISTAR 1-01 (NCT06303505) evaluating advanced NSCLC adenocarcinoma and platinum-resistant ovarian cancer (152, 159, 160).

#### 3.3.2 Folate Receptor Alpha (FRα)

Folate receptor α (FRα) is the most widely expressed of the folate receptor isoforms and helps support biosynthetic pathways such as DNA synthesis, important for rapidly dividing cancer cells (161). In NSCLC, FRα expression is enriched in 74% of lung adenocarcinoma patients compared to only 13% of squamous tumors, making it more ideal for patients with non-squamous lung cancer (162). Farletuzumab ecteribulin (MORAb-202), an FRα-directed ADC carrying an eribulin payload with DAR 4, demonstrated an overall tolerable safety profile (majority TRAEs leukopenia or neutropenia 45% each) in a small phase I trial (22 patients total) (NCT03386942) of refractory FRα-positive solid tumors, though pneumonitis occurred in 14% of patients (163). The planned phase II trial (NCT05577715) in pretreated metastatic NSCLC adenocarcinoma was terminated (164, 165). Other phase I trials involving FRα ADC that include lung cancer patients are LY4170156 (NCT06400472) and ZW191 (NCT06555744). Otherwise, the most advanced FRα ADC is Mirvetuximab soravtansine, which is FDA approved for FRα positive patients with platinum-resistant ovarian cancer, although it remains to be seen if this target is viable in lung cancer patients (166).

#### 3.3.3 ROR2

ROR2, a receptor involved in noncanonical Wnt5a signaling, is aberrantly expressed in multiple malignancies including NSCLC, where retrospective analyses have shown ROR2-high expression in nearly half of tumors compared with minimal levels in adjacent normal lung tissue. Its elevated expression correlates with more advanced disease and independently poorer survival (167, 168). The leading therapeutic candidate, Ozuriftamab vedotin (CAB-ROR2-ADC; BioAtla BA3021), is a conditionally active ADC consisting of a humanized anti-ROR2 IgG1 linked via a protease-cleavable linker to the MMAE payload, engineered to preferentially activate within the acidic tumor microenvironment. Early-phase studies have demonstrated feasible safety and supported phase 2 expansion in ROR2-positive solid tumors, including NSCLC patients who progressed after immune checkpoint inhibitors or targeted therapies, though clear efficacy in lung cancer has not yet been established (169). More promising activity has been observed in other tumors such as head and neck squamous cell carcinoma, where response rates of 36% and disease control of 87% (158) led to FDA Fast-Track designation, leaving open the question of whether similar benefit can be achieved in NSCLC.

## 4 Current challenges of ADCs and antibody-based treatments

Despite ADCs representing one of the most rapidly advancing therapeutic classes in lung cancer, with multiple approvals, their broader and more durable clinical impact is constrained by biological, technical, and translational challenges that have resulted in several ADCs failing to meet primary clinical endpoints. This is most likely due to the fact that an ADC’s success is rarely explained by target expression alone. Rather, successful ADC development generally requires alignment across five main domains: target suitability, efficient internalization/trafficking, payload sensitivity, therapeutic window, and patient-selection strategy. Failure may occur when one or more of these domains are mismatched along with other factors such as limited tumor penetration given their large size (~150 kDA), inadequate intracellular delivery, weak payload susceptibility, toxicity constraints, and failing to capture the biologic states that determine benefit.

These challenges are particularly pronounced in lung cancer due to tumor heterogeneity, limited tissue availability for biomarker testing, cumulative treatment-related toxicities, and the complex pharmacology of ADCs. Addressing these limitations is critical to optimizing ADC performance and defining their role within evolving treatment paradigms.

Currently, the main challenges of ADCs in lung cancer related to target and biomarker selection center on the lack of reliable predictive biomarkers and the difficulty of identifying which patients will benefit most from these therapies. Unlike classic “oncogene-targeted” treatments (such as EGFR or ALK), many ADC targets in NSCLC—such as TROP2, HER3, CEACAM5, MET, and B7-H3—are not binary biomarkers, and their expression is often continuous, heterogeneous, and influenced by prior therapies. Examples of failures include the CEACAM5-directed ADC tusamitamab ravtansine in the phase III CARMEN-LC03 trial (120) which failed despite high CEACAM5 expression by IHC. Diagnostic methods such as IHC face several critical limitations in lung cancer biomarker testing, including technical variability, tissue preservation issues, interpretation challenges, and inadequate predictive value for certain therapies. Recent quantitative immunofluorescence studies demonstrate that all four major ADC targets (HER2, TROP2, HER3, and EGFR) show broad and comparable dynamic ranges of expression, with high proportions of cases above assay limits (170), along with differences in expression of CEACAM5 even between adenocarcinoma and squamous histologies (116) and between primary and metastatic sites (116). Thus, even with high antigen expression, it does not guarantee response, highlighting that antigen expression may be necessary but not sufficient for ADC efficacy, lending to the heterogeneity of biomarker results (171). Another complex challenge for ADCs is that resistance can be multifactorial, with multiple interconnected mechanisms involving the antibody target, intracellular trafficking, payload activity, and the tumor microenvironment (172). Tumors can evade ADCs by reducing or altering antigen availability (target downregulation/loss, clonal selection, of antigen-low subpopulations), which is particularly relevant in NSCLC where target expression is heterogeneous across lesions and can shift over time under therapeutic pressure. Even when antigens remain present, resistance can occur through impaired internalization or altered intracellular trafficking (e.g., increased recycling to the surface or reduced lysosomal processing), preventing payload release (173). A second major resistance axis is payload-related, especially relevant to leading lung ADCs such as the frequently used topoisomerase I inhibitor payloads. After internalization and payload release, tumor cells can acquire resistance through drug-efflux transporters (such as ABC family pumps) that reduce intracellular payload concentration or through intrinsic payload resistance—including changes in topo-I pathway dependence, enhanced DNA damage repair, replication stress response, or anti-apoptotic adaptation (172, 173). This creates potential cross-resistance between ADCs that share payload families (for example, DXd-based ADCs across different targets), which is why sequencing strategies may increasingly require switching payload class or combining ADCs with agents that disrupt DNA repair and represent potential paradigm shifts on how to overcome resistance to ADCs and future management.

Overall, these combined issues have continued to be an ongoing area of interest, especially as it has become increasingly important as more ADCs are approved and developed in choosing the right target and payload, selecting reliable biomarkers, detecting and overcoming resistance, optimizing DAR, exposure, and antigen heterogeneity, and exploring how they can be safely combined with other modalities and systemic therapies.

## 5 Future directions

Ongoing advancements in ADCs have led to numerous potential new targets and therapeutics along with different combinations of ADCs that seek to further improve clinical outcomes for patients with lung cancer. However, other future directions have also been considered, such as whether ADCs are analogous to chemotherapy and will also be able to replace it in the future. Ongoing trials are evaluating ADCs in first-line settings (DESTINY-Lung04 and TROPION-Lung02), potentially replacing chemoimmunotherapy combinations entirely in selected populations.

A previously described challenge with ADCs is target selection when eligibility is determined solely by IHC and other static biomarker measurements. An emerging method to more accurately quantify expression is the use of quantitative image analysis, specifically for TROP2 as a “normalized membrane ratio”. In clinical trials, responses to Dato-DXd occurred regardless of TROP2 expression levels by conventional IHC in both TROPION-PanTumor01 (174) and TROPION-Lung01 (72) trials, leading investigators to explore more sophisticated quantitative approaches. The TROP2 normalized membrane ratio via a computational pathology approach that quantifies and locates targets like TROP2 with whole slide imaging (WSI) analysis and automated image analysis to develop a quantitative continuous scoring (QCS)-NMR model that specifically measures TROP2 expression on the cell membrane and cytoplasm and their optical density, as opposed to total cellular expression using a supervised deep learning algorithm trained on pathologist’s annotations. This methodology uses continuous scoring rather than categorical IHC scoring (0, 1+, 2+, 3+), potentially providing more granular information about the actual target available for antibody binding. This was applied to the TROPION-Lung01 cohorts, in which TROP2 QCS-NMR positivity was predictive of longer PFS when treated with Dato-DXd in the non-squamous and non-actionable genomic altered population with 7.2 months compared to 4.0 months in the TROP2 QCS-NMR negative cohort respectively (175). Currently, there is an ongoing trial, TROPION-Lung17 (NCT07291037), that seeks to validate NMR and the QCS model as a potential new way to evaluate TROP2 as a biomarker and lead to other biomarkers being evaluated in a similar fashion. Other emerging features have included the use of antibody- and ligand-based PET imaging such as in radiotheranostics, which have the potential to measure whole-body antigen expression, capture spatial and temporal heterogeneity, and predict ADC uptake and response along with monitoring early resistance and toxicity (30, 176). Several targets such as CEACAM6 (CD66c) (177), DLL3 (178), and Nectin-4 (179) have demonstrated preliminary uses in lung cancer as they further expand in utility.

Lastly, due to the advancement of antibody technology since the advent of ADCs, there are now multiple variations of antibody modalities. There is a growing trend of expanding not to just single antibody targets but to multiple targets to increase efficacy and broaden the various pathways and resistance mechanisms for lung cancer patients. Currently, the most advanced of these strategies is utilizing two antibodies or bispecifics and whether it is linked with a toxic payload or not. Examples of these bispecifics range from already approved mechanisms such as Amivantamab (180), a bispecific antibody targeting EGFR and MET, Zenocutuzumab, a bispecific against HER3 and HER2 (181), and Tarlatamab, a bispecific T-cell engager (BiTE) that targets DLL3 and CD3 (182). This concept has now not only expanded into immunotherapy targets such as in Ivonescimab, a bispecific with PD-1 and VEGF, but also ADCs such as Izalontamab brengitecan, an EGFR-HER3 bispecific ADC (183, 184) with promising activity in *EGFR* mutant NSCLC. An even more novel design can be seen with biparatopic ADCs that target two distinct epitopes on the same antigen, enabling higher binding potential and receptor blockade and increased payload delivery, with its first approval with Zanidatamab (HER2 x HER2 bispecific) approved for HER2-positive biliary tract cancer (185) and with the potential of applying these concepts to other tumor types. Despite early promising preclinical results (186) from REGN5093-M114, a biparatopic ADC targeting MET x MET with a linker-payload (M114, carrying the maytansine derivative M24, a potent inhibitor of microtubule assembly), it demonstrated disappointing results during its phase I/II trial (NCT04982224) and is no longer in development. As this technology continues to evolve, it remains to be seen if it will be able to overcome the limitations of traditional ADCs (summarized in **Table 3**).

**Table 3:** Examples of novel antibody designs that involve patients with lung cancer.

Type of design	Examples	Targets	Clinical trials (phase)
Bispecific Antibodies	Amivantamab	EGFR x MET	MARIPOSA (Phase III) NCT04487080 (180)
	Zenocutuzumab	HER3 x HER2	eNRGy (Phase II) NCT02912949 (181)
	Izalontamab	EGFR x HER3	NCT04603287 (187) (Phase I/Ib)
	Pamvatamig (MCLA-129)	EGFR x MET	NCT04930432 (188) (Phase I/II)
	Ivonescimab	PD-1 x VEGF	HARMONi-A (Phase III) NCT05184712 (189) HARMONi-2 (Phase III) NCT05499390 (190)
	BNT327/PM8002	PD-1 x VEGF-A	NCT06712355 (191) (Phase III)
	PM8002	PD-L1 x VEGF-A	NCT05918445 (192) (Phase Ib/II)
	Volrustomig	PD-1 x CTLA-4	eVOLVE-Lung02 (Phase III) NCT05984277 (193)
	Cadonilimab	PD-1 x CTLA-4	NCT05816499 (194) (Phase I/II)
	KN046	PD-L1 x CTLA-4	NCT04054531 (195) (Phase II)
	Tobemstomig	PD-1 x LAG-3	NCT05775289 (196) (Phase II)
	Rilvegostomig	PD-1 x TIGIT	ARTEMIDE-01 (Phase I/II) NCT04995523 (197)
	Sabestomig (AZD7789)	PD-1 x TIM-3	NCT04931654 (198) (Phase I/II)
Bispecific ADCs	Izalontamab brengitecan	EGFR x HER3	NCT05194982 (199) (Phase Ia/Ib)
	AZD9592	EGFR x MET	NCT05647122 (200) (Phase I)
Biparatopic ADCs	REGN5093-M114	MET x MET	NCT04982224 (201) (Phase I/II)
Bispecific and trispecific T-cell engagers (BiTEs and TriTEs)			
BiTEs	Tarlatamab	DLL3 x CD3	DeLLphi-304 (Phase III) NCT05740566 (202)
TriTEs	Gocatamig (MK-6070/HPN328)	DLL3 x CD3 x albumin	NCT06780137 (203) (Phase I/II)
Immune-stimulating ADCs (ISAC)	TAK-500	CCR2 antibody + STING agonist TAK-500	NCT05070247 (204) (Phase I/II)
	BDC-1001	HER2 + TLR7/8 agonist	NCT04278144 (205) (Phase I/II)
	PF-08046037 (206)	PD-L1 + TLR7 agonist payload	NCT06974734 (Phase I)
Probody Drug Conjugate (PDC)	Praluzatamab ravtansine	CD166	PROCLAIM-CX-2009 (207) (Phase I/II) NCT03149549
	CX-2029	CD71	NCT03543813 (208) (Phase I/II)
Protein-degrader ADCs (DACs)	ORM-5029	HER2 + GSPT1 degrader	NCT05511844 (209) (PhaseI)
	DAC-1522 (210)	TROP2	Preclinical (no trials yet)
	84-EBET (211)	CEACAM6 and BET degrader	Preclinical (no trials yet)

## 6 Conclusion

Just as advances in antibody technologies drove a paradigm shift in cancer treatment, first with PD-1/PD-L1 inhibitors in immunotherapy and then with targeted therapies, ADCs are now introducing new possibilities for the selective, tumor-targeted delivery of cytotoxic agents. This has also introduced exciting opportunities for further expanding this concept to other mechanisms of action and repertoires of targets and pushing the boundaries of further clinical improvement. However, ADCs must still be applied in biomarker-defined populations in lung cancer. Current ADC approvals are mainly driven by selective groups of oncogenic drivers and have demonstrated their ability as a monotherapy in unselected lung cancer patient populations and broader prior immunotherapy-treated patients. Additionally, further research is needed on why some ADCs fail and others succeed, analyzing whether it is related to their targets, biomarker analysis, or their payload-linker systems. Moreover, trials are needed to confirm these innovative therapeutics and how they will continue to fit into the paradigm management of lung cancer; many trials are already looking forward to the prospects of using them earlier and with more inclusive groups of patients.
